# GBE50 Attenuates Inflammatory Response by Inhibiting the p38 MAPK and NF-****κ****B Pathways in LPS-Stimulated Microglial Cells

**DOI:** 10.1155/2014/368598

**Published:** 2014-03-24

**Authors:** Gai-ying He, Chong-gang Yuan, Li Hao, Ying Xu, Zhi-xiong Zhang

**Affiliations:** ^1^Department of Physiology, Basic Medical College, Shanghai University of Traditional Chinese Medicine, Shanghai 201203, China; ^2^Department of Physiology, College of Life Science, East China Normal University, Shanghai 200062, China; ^3^Department of Anatomy, Basic Medical College, Henan University of Traditional Chinese Medicine, Zhengzhou 450008, China

## Abstract

Overactivated microglia contribute to a variety of pathological conditions in the central nervous system. The major goal of the present study is to evaluate the potential suppressing effects of a new type of Ginko biloba extract, GBE50, on activated microglia which causes proinflammatory responses and to explore the underlying molecular mechanisms. Murine BV2 microglia cells, with or without pretreatmentof GBE50 at various concentrations, were activated by incubation with lipopolysaccharide (LPS). A series of biochemical and microscopic assays were performed to measure cell viability, cell morphology, release of tumor necrosis factor-**α** (TNF-**α**) and interleukin-1**β** (IL-1**β**), and signal transduction via the p38 MAPK and nuclear factor-kappa B (NF-**κ**B) p65 pathways. We found that GBE50 pretreatment suppressed LPS-induced morphological changes in BV2 cells. Moreover, GBE50 treatment significantly reduced the release of proinflammatory cytokines, TNF-**α** and IL-1**β**, and inhibited the associated signal transduction through the p38 MAPK and NF-**κ**B p65 pathways. These results demonstrated the anti-inflammatory effect of GBE50 on LPS-activated BV2 microglia cells, and indicated that GBE50 reduced the LPS-induced proinflammatory TNF-**α** and IL-1**β** release by inhibiting signal transduction through the NF-**κ**B p65 and p38 MAPK pathways. Our findings reveal, at least in part, the molecular basis underlying the anti-inflammatory effects of GBE50.

## 1. Introduction

Microglia (MG) cells are a major type of immunoreactive cells in the central nervous system (CNS) and account for about 5–20% of the total glial cells. Overactivated MG cause serious neuronal damage [1] and lead to abnormal immune responses in neurodegenerative diseases [2]. Various autoimmune responses caused by hyperactivated MG are believed to contribute to neuronal death and brain injury. Activated MG release proinflammatory and/or cytotoxic factors such as IL-1, IL-6, TNF-*α*, NO, and ROS [3–5] that damage neurons in the CNS. IL-1*β* was reported to increase neuronal cholinesterase expression and enzyme activity causing CNS cholinergic system dysfunction and glial cells in mixed culture to release an excitatory neurotoxin glutamate that leads to oligodendrocyte cell death [6, 7]. IL-6 binds to hippocampal neuron N-methyl-D-aspartate receptors (NMDA R) which causes neuronal Ca^2+^ influx by the activation of the JAKs/STATs pathway and leads to overphosphorylation of the Tau protein that promotes nerve fiber tangles (NFT) formation [8]. In Alzheimer's disease (AD), the interaction of TNF-*α* and A*β* promotes each other's secretion, forming a vicious cycle and aggravating the pathological changes observed in AD [9]. Activated MG release not only cytokines, but also adhesion molecules and chemokines. All of these factors are able to cause neuronal damage and apoptosis that lead to further activation of the MG and a vicious cycle that further exacerbates the neuronal injury [10].

*Ginkgo biloba* extract 50 (GBE50) is a new type of ginkgo leaf extract that has been patented in the USA and other countries. GBE50 contains 44.1% ginkgo-flavone glycosides (including kaempferol, quercetin, and isorhamnetin derivatives) and 6.4% lactones (including ginkgolides A, B, C, and J and bilobalide), and the adverse ingredient ginkgo acid content is smaller than 5 ppm. Some studies have shown that GBE50 has antioxidative and anti-inflammatory effects [11, 12] and has been used in the prevention or treatment of cardiovascular diseases in aging populations. However, little is known about the role of GBE50 in the nervous system. This study investigated the anti-inflammatory effects of pretreatment with GBE50 on LPS-induced murine BV2 microglial cells. Our results provide an experimental basis for the anti-inflammatory application of GBE50 in combating neuronal diseases.

## 2. Materials and Methods

### 2.1. Drugs and Reagents

GBE50 and EGB761 were kindly provided by Shanghai Xing Ling Technology Pharmaceutical Co., Ltd. (Shanghai, China). Dulbecco's modified Eagle's medium (DMEM), fetal calf serum (FCS), horse serum (HS), and penicillin-streptomycin were purchased from Gibco (Grand Island, NY, USA). Paraformaldehyde and LPS were purchased from Sigma (St. Louis, MO, USA). The murine specific enzyme-linked immunosorbent assay (ELISA) kit was purchased from R&D Systems (Minneapolis, MN, USA). Rabbit anti-rat antibodies against p38 MAPK, phospho-p38 MAPK, NF-*κ*B p65, I*κ*B*α*, and *β*-tubulin were purchased from Cell Signal Technology (Beverly, MA, USA). Alexa 488 donkey anti-rabbit IgG was purchased from Jackson ImmunoResearch Laboratories (West Grove, PA, USA). The BCA protein assay kit and enhanced chemiluminescence (ECL) detection system were purchased from Beyotime (Shanghai, China).

### 2.2. Cell Culture and Treatments

The mouse BV2 microglial cells were purchased from the Chinese Academy of Medical Sciences, Beijing, China. BV2 cells were grown at 37°C in 5% CO_2_ in DMEM (high glucose) supplemented with 10% FCS and 1% penicillin-streptomycin, and the media were changed every 2 or 3 days. Adherent cells at the logarithmic growth phase were digested by D-Hank's solution containing 0.25% trypsin, seeded, and cultured for 24 h before treatments. The cells were pretreated with GBE50 at 0.01, 0.1, 1, 10, and 100 *μ*g/mL or with EGB761 at 10 *μ*g/mL for 2 h and then activated with 1 *μ*g/mL LPS for 22 h. The BV2 cells treated only with solvent for 24 h and only with LPS were used as controls and the model.

### 2.3. MTT Assay

Cultured BV2 cells in 96-well dish (1 × 10^4^ cells/well) were treated with GBE50 at 0.01, 0.1, 1, 10, and 100 *μ*g/mL or with 10 *μ*g/mL EGB761 for 2 h and then exposed to LPS (1 *μ*g/mL) for 22 h. Then 20 *μ*L of MTT solution (5 mg/mL) was added to each well and cells were incubated for additional 4 h at 37°C. Subsequently, the medium was removed and the crystals were dissolved in 150 *μ*L of dimethyl sulfoxide (DMSO). The cell viability was quantified by measuring the optical density (OD) at 570 nm using a microplate reader.

### 2.4. Morphological Changes of LPS-Activated BV2 Cells

Cultured BV2 cells in 24-well dish (5 × 10^4^ cells/well) were treated with GBE50 at 0.01, 0.1, 1, 10, and 100 *μ*g/mL or with EGB761 (10 *μ*g/mL) for 2 h and then exposed to LPS (1 *μ*g/mL) for 22 h. The control group was treated with a solvent for 24 h. After treatment, the medium was removed, and the cells were fixed with 4% formaldehyde for 15 mins at room temperature. Fixed BV2 cells were washed with 0.01 M phosphate-buffered saline (PBS) twice for 10 mins, and the cell morphology was observed using a light microscope.

### 2.5. ELISA Assay

BV2 cells were seeded in 24-well dish (2 × 10^4^ cells/well). Cells were then incubated with GBE50 or EGB761 for 2 h before treatment with LPS (1 *μ*g/mL) for 22 h. The control group was only treated with the same amount of solvent for 24 h. The cell culture medium was collected and used to measure TNF-*α* and IL-1*β* levels by using murine specific ELISA kits according to the manufacturer's suggested protocol.

### 2.6. Immunofluorescence Staining

BV2 cells were seeded in a 24-well dish (3 × 10^4^ cells/well) and cultured for 24 h. The cells were then incubated with GBE50 or EGB761 for 2 h before treatment with LPS (1 *μ*g/mL) for 22 h. The control group was treated only with the same amount of solvent for 24 h. The cells were fixed with 4% paraformaldehyde in 0.1 M phosphate buffer (PB) for 20 mins at room temperature. After rinsing with 0.01 M PBS, the cells were fixed and permeabilized with 0.2% Triton X-100 in 0.01 M PBS for 30 mins. Cells were washed three times with 0.01 M PBS (5 mins/wash), blocked with 5% horse serum at room temperature for 30 mins, and incubated with rabbit an anti-mouse NF-*κ*B p65 antibody (1 : 50 dilution) overnight at 4°C. In the following day, cells were incubated with the secondary antibody DyLight 488 donkey anti-rabbit IgG (1 : 100 dilution) for 2 h at room temperature, washed three times with 0.01 M PBS (15 mins/wash), and incubated with 100 ng/mL DAPI for 3 mins. All images were captured with a fluorescence microscope (Leica). Results presented here show representative images from three independent experiments.

### 2.7. Immunoblot Analysis

BV2 cells of different groups were lysed with ice-cold RIPA lysis buffer and phenylmethylsulfonyl fluoride (PMSF) to extract cytoplasmic proteins. The protein concentrations of cell lysates were determined by bicinchoninic acid (BCA) assay. Equal amounts of total cellular protein (20 *μ*g per lane) were used for 12% SDS-polyacrylamide gel electrophoresis (SDS-PAGE) and transferred onto immunoblot polyvinylidene difluoride (PVDF) membranes. The membranes were blocked with 5% nonfat milk in Tris-buffered saline containing 0.1% Tween 20 (TBST) for 1 h and incubated with rabbit anti-mouse antibodies against NF-*κ*Bp65, p38 MAPK, P-p38 MAPK, I*κ*B*α* (1 : 1,000 dilution), or *β*-tubulin (1 : 3,000 dilution) overnight at 4°C. The membranes were then washed three times (5 mins/wash) with TBST and incubated with a 1 : 2,000 dilution of horseradish peroxidase-conjugated secondary antibodies for 2 h at room temperature. The blots were again washed three times (15 mins/wash) in TBST and developed using the ECL detection system (A : B = 1 : 1) for 2–5 mins at room temperature. Normalized bands densities were analyzed using the Gel-Pro Analyzer software and expressed as ratios to *β*-tubulin.

### 2.8. Statistical Analysis

All data are presented as mean ± standard deviation (SD). SPSS 17.0 software was used to perform statistical analyses. Differences among means were measured by one-way analysis of variance (ANOVA) for multiple comparisons. A *P* < 0.05 was considered significant and *P* < 0.01 was considered highly significant.

## 3. Results

### 3.1. The Effect of GBE50 on the Cell Viability of BV2 Cells

**Figure 1:**
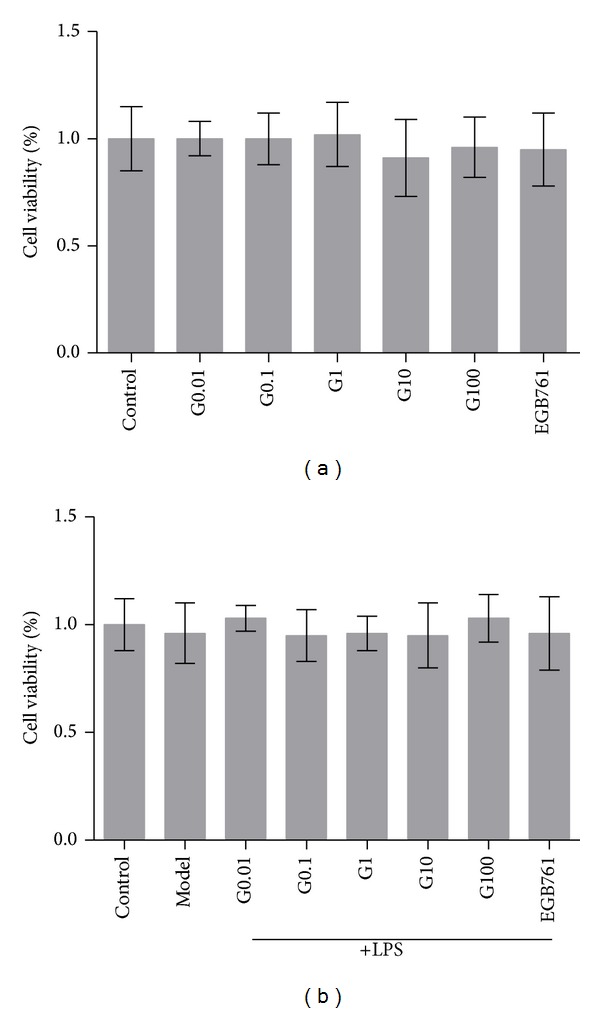
The effect of GBE50 on viability of BV2 cells. (a) The effect of various GBE50 concentrations on BV2 cell viability. No significant difference was observed (*P* > 0.05). (b) The effect of different concentrations of GBE50 on BV2 cell viability activated by LPS. No significant difference was observed (*P* > 0.05).

Since the influence of GBE50 on the cell viability of BV2 cells was unclear, we first performed an MTT assay to compare the cell viability of BV2 cells treated with or without GBE50. Our results showed that there were no significant differences between the control group and GBE50 groups treated with various concentrations (Figure 1(a), *n* = 6, *P* > 0.05). These data suggest that GBE50 does not significantly alter the cell viability of BV2 cells within the concentration range.

### 3.2. The Effect of GBE50 on the Cell Viability of LPS-Activated BV2 Cells

We tested whether GBE50 altered the cell viability of LPS-activated BV2 cells. There was no significant change in the cell viability among each group (Figure 1(b), *n* = 6, *P* > 0.05). These data indicate that GBE50 had no significant effect on the cell viability of LPS-activated BV2 cells.

### 3.3. The Effect of GBE50 on the Morphology of LPS-Activated BV2 Cells

**Figure 2:**
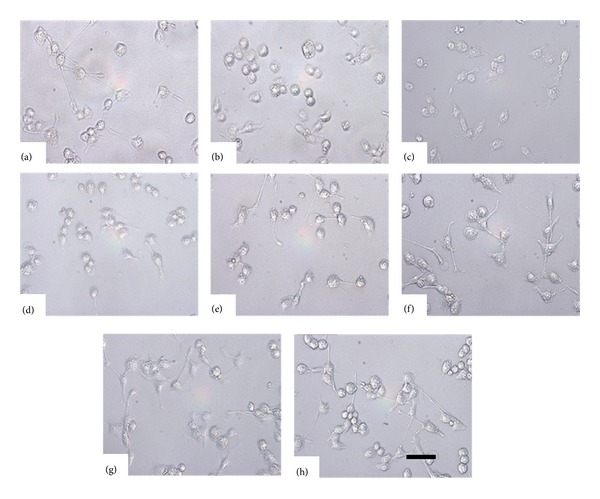
The effect of GBE50 on the morphology of BV2 cells between treated and untreated groups. (a) The morphology of BV2 cells in the control group. (b) The morphology of BV2 cells activated by LPS. (c)–(g) The effect of GBE50 at indicated concentrations on the morphology of BV2 cells activated by LPS. (h) The effect of EGB761 (10 *μ*g/mL) on the morphology of BV2 cells activated by LPS. All images were low magnification (10x objective lens) photomicrographs of unstained BV2 cells. Scale bar = 50 *μ*m.

The morphological changes of LPS-activated BV2 cells with or without GBE50 pretreatment were analyzed using a Zeiss microscope. As shown in Figure 2, BV2 cells in the control group exist in a resting state, with smaller bodies and longer pseudopodia (Figure 2(a)), while LPS-activated BV2 cells exist in an amoeba-like state, with larger cell bodies and shorter branch (Figure 2(b)). When BV2 cells were pretreated with increased concentrations of GBE50 and then exposed to LPS (Figures 2(c)–2(g)), the morphology of BV2 cells was more similar to that observed during the resting state, with smaller cell bodies and longer branches. The morphology of the EGB761 treated cells was similar to that observed for the control group (Figure 2(h)). These results suggest that GBE50 can suppress LPS-stimulated BV2 cells' activation.

### 3.4. The Effect of GBE50 on TNF-*α* and IL-1*β* Released by LPS-Activated BV2 Cells

**Figure 3:**
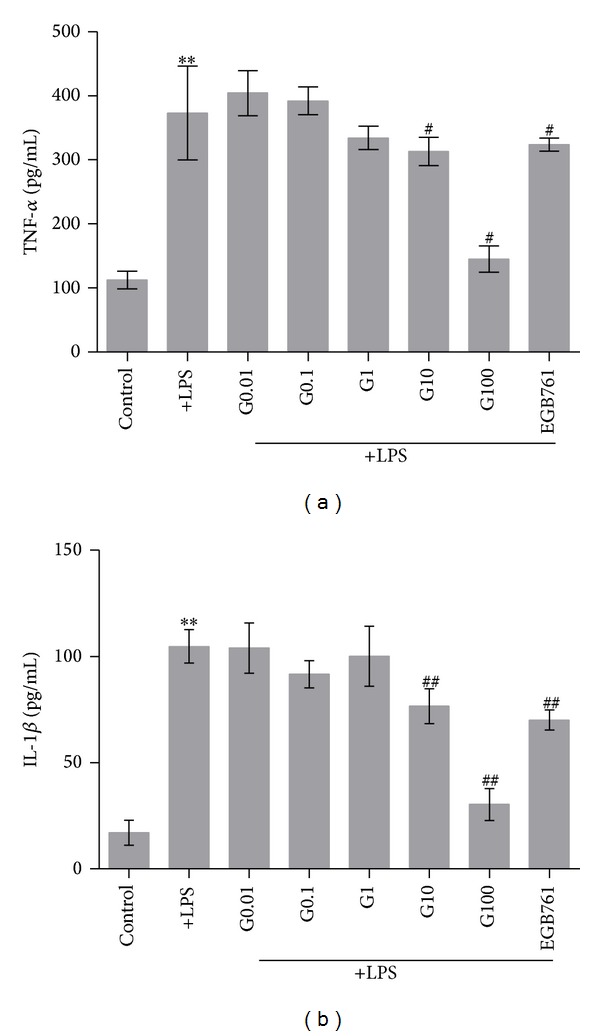
The effect of GBE50 on TNF-*α* and IL-1*β* in BV2 cells activated by LPS. (a) The effect of GBE50 on TNF-*α* in BV2 cells activated by LPS. GBE50 significantly reduced TNF-*α* at doses of 10 and 100 *μ*g/mL. (b) The effect of GBE50 on IL-1*β* in BV2 cells activated by LPS, GBE50 significantly reduced IL-1*β* at doses of 10 and 100 *μ*g/mL. *P < 0.05, **P < 0.01 versus control and ^#^ *P* < 0.05, ^##^ *P* < 0.01 versus LPS group.

The content of TNF-*α* and IL-1*β* was measured by ELISA. As shown in Figure 3, LPS-activated BV2 cells showed a significant increase in TNF-*α* and IL-1*β*. Pretreating cells with GBE50 at 10 or 100 *μ*g/mL significantly reduced TNF-*α* and IL-1*β* secretion; a similar effect was observed for the EGB761 treated group (Figures 3(a) and 3(b)). These results reveal that GBE50 can reduce the level of TNF-*α* and IL-1*β* proinflammatory cytokines released by activated BV2 cells in a dose-dependent manner.

### 3.5. The Effect of GBE50 on NF-*κ*B p65 Nuclear Translocation in LPS-Activated BV2 Cells

**Figure 4:**
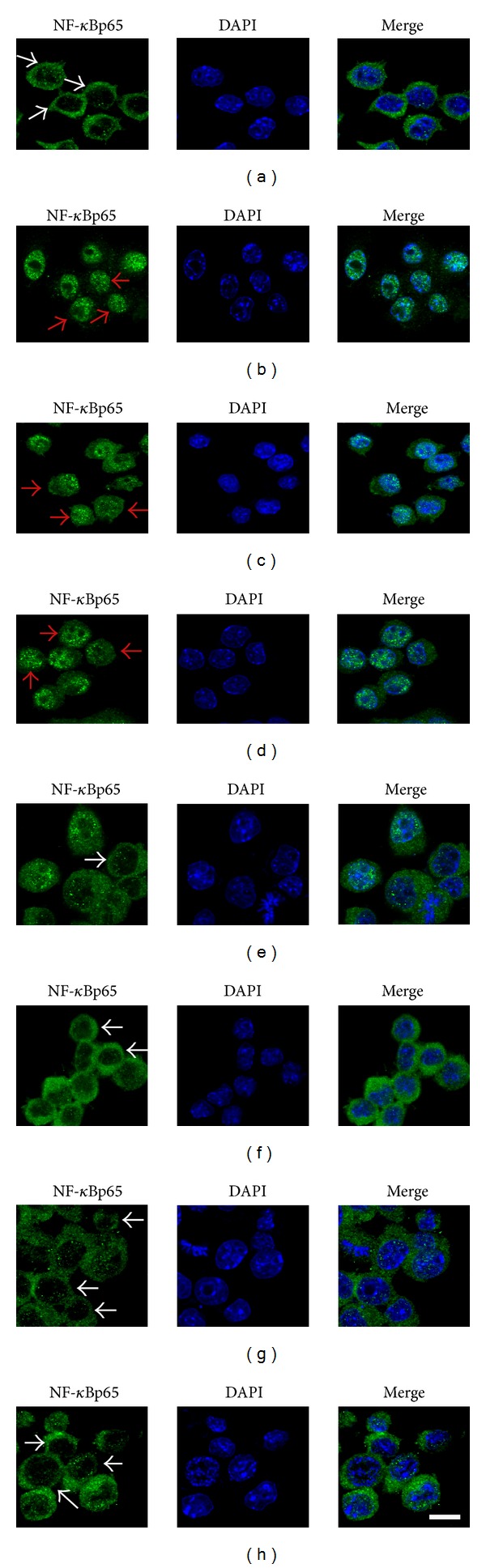
The effect of GBE50 on NF-*κ*B p65 nuclear translocation in LPS-activated BV2 cells. (a) There is almost no expression of NF-*κ*B p65 in the nucleus of the control group. (b) In the LPS group, there is almost complete translocation of NF-*κ*B p65 from the cytoplasm to the nucleus. (c)–(g) NF-*κ*Bp65 nuclear translocation was gradually inhibited at GBE50 concentrations of 1, 10, and 100 *μ*g/mL. (h) EGB761 also had the obvious inhibitory effects on NF-*κ*B p65 nuclear translocation. Confocal microscopy images of BV2 cells were stained with an antibody against NF-*κ*B p65 (green) and counterstained with DAPI (blue) to label nuclei. Confocal images were captured through the center of control and WAVE2-KD acini immunostained for E-cadherin (green) and counterstained with Alexa-568 phalloidin (red) to label actin filaments and DAPI (blue) to label nuclei. White arrowheads showed that NF-*κ*B p65 mainly localized in the cytoplasm and red arrowheads indicated the nuclear translocation of NF-*κ*B p65. Scale bar = 10 *μ*m.

The NF-*κ*B signaling pathway has been implicated in LPS-induced microglial activation and production of proinflammatory cytokines [5]. To understand the molecular mechanism of the anti-inflammatory effect of GBE50, we analyzed the activation of the NF-*κ*B signaling pathway by measuring the NF-*κ*B p65 nuclear translocation. As shown in Figure 4, activation of BV2 cells resulted in almost the complete translocation of NF-*κ*B p65 from the cytoplasm to the nucleus (Figure 4). Pretreatment of cells with GBE50 showed a dose-dependent influence on NF-*κ*Bp65 nuclear translocation. The inhibitory effects were clearly observed when GBE50 concentrations were at 1, 10, and 100 *μ*g/mL; EGB761 treated cells also demonstrated notable inhibitory effects (Figure 4). These data indicate that GBE50 treatment can reduce NF-*κ*B p65 activation by inhibiting the translocation of NF-*κ*B p65 from the cytoplasm to the nucleus.

### 3.6. The Effect of GBE50 on NF-*κ*B p65 and I*κ*B*α* Protein Expression in LPS-Activated BV2 Cells

**Figure 5:**
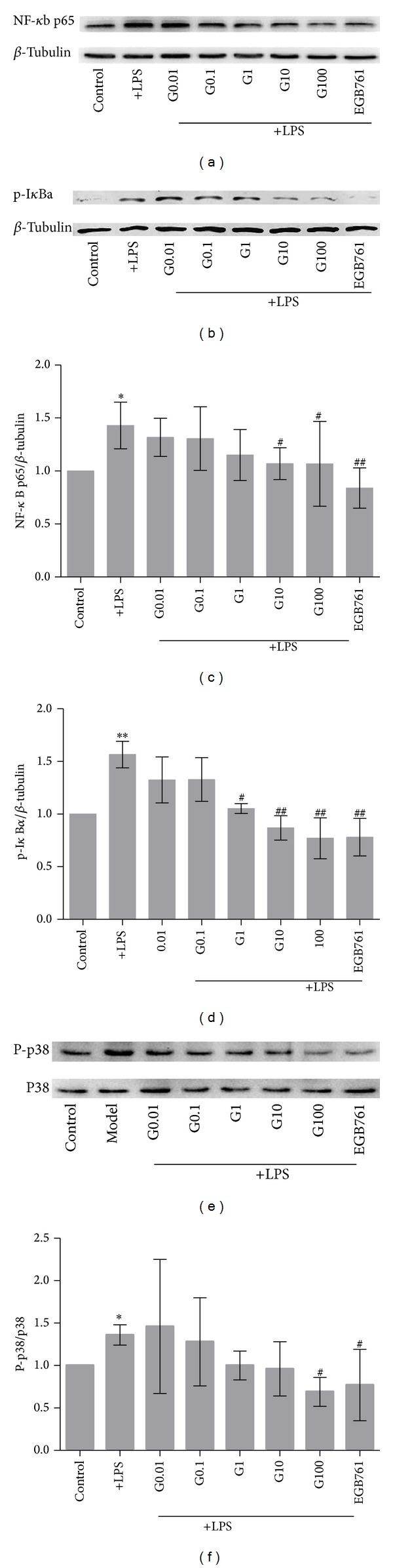
The effect of GBE50 on NF-*κ*B p65, I*κ*B*α*, and P-p38/p38 of BV2 cells activated by LPS. (a) The effect of GBE50 on a representative blot of NF-*κ*B p65 protein expression in BV2 cells activated by LPS. (b) The effect of GBE50 on NF-*κ*B p65 between treated and untreated groups. (c) The effect of GBE50 on a representative blot of the ratio of I*κ*B*α* in BV2 cells activated by LPS. (d) The effect of GBE50 on the ratio of I*κ*B*α* between treated and untreated groups. (e) The effect of GBE50 on a representative blot of the ratio of P-p38/p38 in BV2 cells activated by LPS. (f) The effect of GBE50 on the ratio of P-p38/p38 between treated and untreated groups. *P < 0.05, **P < 0.01 versus control and ^#^ *P* < 0.05, ^##^ *P* < 0.01 versus LPS group.

The NF-*κ*B p65 protein expression levels in each group were measured by immunoblot analysis. LPS activation significantly increased NF-*κ*B p65 expression. Pretreatment of GBE50 at 0.01 or 0.1 *μ*g/mL did not significantly alter the NF-*κ*B p65 expression level. However, when GBE50 concentration was raised to 10 or 100 *μ*g/mL, NF-*κ*B p65 and I*κ*B*α* protein expression were reduced significantly, which were similar to that observed for the EGB761 treated group (*P* < 0.05 ~ 0.01) (Figures 5(a)–5(d)).

### 3.7. The Effect of GBE50 on P38 Phosphorylation in LPS-Activated BV2 Cells

The p38 MAPK signaling pathway was also implicated in LPS-induced BV2 cells activation [5]. To explore the potential changes of the p38 MAPK signaling pathway caused by GBE50 pretreatment, we measured the p38 protein and P-p38 protein expression levels by immunoblot analysis. We found that LPS and GBE50 did not significantly alter the p38 protein expression level (Figure 5(e)). However, the P-p38 of BV2 cells was significantly increased by LPS, and GBE50 pretreatment suppressed P-p38 in a dose-dependent manner (Figure 5(c)). When the GBE50 concentration was 100 *μ*g/mL, the P-p38/total p38 ratio was significantly reduced compared with LPS group (*P* < 0.05) (Figure 5(f)). Taken together, our findings indicate that GBE50 can suppress the phosphorylation of p38.

## 4. Discussion

Recent studies have demonstrated that GBE has an extensive role in the CNS. GBE could regulate cholinergic function, act as an antioxidant and scavenge-free radicals [13], promote the recovery from nerve cell damage, and slow any dementia caused by a decline in cognitive ability. Additionally, GBE could reduce the expression of nitric oxide synthase (NOS) [14], IL-1*β* [15], cyclooxygenase-2 (COX-2), TNF-*α* [16], and IL-6 [17].

EGB761 is a standard extract from the leaves of* Ginkgo biloba* containing 24% ginkgo-flavone glycosides and 6% terpene lactones. The GBE50 used in our study is a new type of* Ginkgo biloba* extract developed independently and patented in multiple nations. GBE50 has more ginkgo-flavone glycosides and terpene lactones than EGB761. Our previous studies showed that GBE50 prevented age-related learning and memory impairment and reduced the expression of several proinflammatory cytokines, including IL-1*β* and IL-6, in hippocampal cells [12, 18].

Microglia are a major type of inflammatory cells in the CNS [19, 20], which exist mainly in two forms: resting and amoeba-like states. In normal conditions, the microglia cells are in a resting state; however, specific stimuli, such as infection, activate these cells, causing their morphology to change into the amoeba-like state [21, 22]. In this study, we observed that GBE50 suppressed LPS-induced morphological changes in BV2 cells, which indicated that GBE50 inhibited microglial activation induced by LPS.

TNF-*α* and IL-1*β* are two important proinflammatory cytokines that lead to widespread toxicity in the CNS. TNF-*α* derived from LPS-activated microglia plays a crucial role, not only in apoptosis but also during inflammatory and immune responses. TNF-*α* causes the activation of microglia, promotes the expression of IL-1*β*, IL-6, and iNOS, and also induces TNF-*α* production. Moreover, LPS-activated microglia express more TNF-*α* than microglia treated with TNF receptor 1 (TNFR1)—specific antagonist. These findings suggest that the TNF-*α* produced by BV2 cells involves an autocrine mechanism [23]. TNF-*α* binding to the tumor necrosis factor receptor (TNFR) in neurons regulates downstream apoptotic cascades [24]. Stellwagen and Malenka [25] reported that synaptic injury is mediated by glial TNF-*α*. IL-1*β*, another major proinflammatory cytokine, induced neuronal and synaptic damage. In the inflamed hippocampus, IL-1*β* and IL-1RI were expressed mainly in microglia and neurons, respectively. IL-1*β* inhibits N-methyl-D-aspartate- (NMDA-) induced outward currents through p38 MAPK signaling and increases the excitability of hippocampal neurons [26]. Rossi and Tanaka [27, 28] confirmed that IL-1*β* caused synaptic hyperexcitability in multiple sclerosis and induced Parkinson's disease. When such activated microglia were placed in coculture with primary neocortical neurons, a significant increase in neuronal tau phosphorylation was accompanied by a decline in synaptophysin levels [29]. IL-1*β* activated microglia secreted increased amounts of proinflammatory cytokines, which resulted in greater damage to the CNS.

In this study, TNF-*α* and IL-1*β* were reduced by pretreating cells with GBE50 in a dose-dependent manner. These findings suggested that GBE50 could significantly reduce proinflammatory cytokine release and prevent neuronal damage in the CNS.

The expression of proinflammatory cytokines and other harmful signaling molecules is regulated by p38 MAPK and NF-*κ*B pathways in the CNS [30–32]. LPS-induced microglial cell activation and production of proinflammatory mediators IL-6, IL-1*β*, and TNF-*α* are regulated by NF-*κ*B signaling pathway and phosphorylation of MAPKs (ERK, p38, and JNK) [5]. Additionally, the activation of the MAPK/NF-*κ*B signaling pathway also generates ROS that may contribute to neuronal damage [4]. Involvement of the p38 MAPK pathway in generating anti-inflammatory cytokines and an inflammatory response in the CNS are also supported by studies with the p38 MAPK inhibitor. Liu et al. [33] found that LPS induced overproduction of nitric oxide synthase (iNOS) in microglia and the expression of iNOS was reduced if the cells were pretreated with a p38 MAPK inhibitor. Wilms and other researchers [34] showed that injection of alpha-synuclein protofibrils into the substantia nigra of adult rats led to a profound activation of microglia and adjacent neuronal cell loss, which could be attenuated by the MAP kinase inhibitor. These findings supported a role for the p38 MAPK pathways in neurotoxicity caused by activated microglia.

In addition to p38 MAPK inhibitor, the NF-*κ*B inhibitor also showed an effect on reducing the release of proinflammatory cytokines and other harmful signaling molecules from activated microglia. Wang et al. [35] observed that saturated fatty acids could initiate microglial activation and stimulate the TLR4/NF-*κ*B pathway to trigger the production of proinflammatory mediators such as TNF-*α*, IL-1*β*, IL-6, and NO, and these effects could be attenuated by an NF-*κ*B inhibitor. Therefore, the activation of the NF-*κ*B p65 and p38 MAPK pathways was involved in the production of proinflammatory cytokines in activated microglia.

Consistent with the involvement of the NF-*κ*B p65 and p38 MAPK pathways in proinflammatory responses, in activated microglia, we found that GBE50 reduced LPS-induced NF-*κ*B p65 expression and nuclear translocation, which were accompanied by parallel reductions in the degradation of I*κ*B*α*. Furthermore, our western blot results revealed that GBE50 attenuated LPS-induced phosphorylation of p38 protein. These findings suggest the anti-inflammatory effects of GBE50 on activated microglia.

Taken together, these data from our present study showed that GBE50 inhibited the LPS-induced I*κ*B*α*/NF-*κ*B p65 and p38 MAPK signal transduction, reduces the release of proinflammatory TNF-*α* and IL-1*β* from LPS-activated microglia cells, and suppresses the LPS-induced microglia activation. These findings provide an experimental and theoretical basis for the further examination of GBE50 as an anti-inflammatory agent in CNS.
